# The effectiveness of an internet-based support program on maternal self-efficacy, postpartum depression and social support for primiparous women during the COVID-19 pandemic: Randomized controlled trial

**DOI:** 10.3389/fpubh.2023.1035872

**Published:** 2023-02-09

**Authors:** Yuting Zhang, Jiemin Zhu, Sen Li, Lingling Huang, Qiyu Fang, Xujuan Zheng

**Affiliations:** ^1^School of Nursing, Health Science Centre, Shenzhen University, Shenzhen, China; ^2^Department of Nursing, School of Medicine, Xiamen University, Xiamen, China; ^3^Department of Obstetrics and Gynecology, Peking University People's Hospital, Beijing, China

**Keywords:** randomized controlled trial, internet-based intervention, primiparous women, maternal self-efficacy, postpartum depression, social support

## Abstract

**Background:**

Many primiparous women usually encounter various parenting and mental health issues after childbirth. The effects of intervention based on internet platform on parenting and mental health outcomes for Chinese first-time mothers remain unknown during the COVID-19 pandemic. Therefore, our research aimed to evaluate the effectiveness of an internet-based support program (ISP) on maternal self-efficacy (MSE), postpartum depression (PPD) and social support for primiparous women amid the pandemic.

**Methods:**

A multicenter randomized controlled trial (RCT) was conducted. From May 2020 to March 2021, 242 primiparous women were recruited in the maternity wards of two hospitals in Shenzhen City, China and randomly assigned to the intervention group and the control group. Women in control group (*n* = 118) received the routine postpartum care, and women in intervention group (*n* = 118) accessed to the ISP intervention (expert education and peer support) and routine postpartum care. Intervention outcomes were measured at baseline before randomization (T0), post-intervention (T1), and three-month follow up (T2) through questionnaires. The chi-square (χ^2^), the independent sample t-test and the repeated measures multivariate analysis of covariance were performed, and the two-tailed p-value <0.05 was regarded as statistically significant.

**Results:**

In comparison with women in the control group, women in the intervention group had a significantly higher score of MSE at T1 (mean: 73.53, standard deviation [SD]: 6.21) and at T2 (mean: 72.90, SD: 6.73); and a lower score of PPD at T1(mean: 6.03, SD: 2.50) and T2 (mean: 5.70, SD: 2.23); and a higher score of social support at T1 (mean: 45.70, SD: 3.73), but no significant difference at T2 (mean: 42.90, SD: 3.29).

**Conclusions:**

The effect of ISP was evaluated to significantly increase the levels of MSE, social support, and to alleviate PPD symptoms for Chinese first-time mothers. As an effective and easily accessible intervention, ISP could become a significant source for health professionals to support primiparous women on parenting and mental health during the COVID-19 pandemic.

**Trial registration:**

The trial is registered at the Chinese Clinical Trials Registry (ChiCTR2000033154).

## Introduction

In March 2020, the WHO had declared the novel COVID-19 as a pandemic worldwide (1). In order to limit the spread of COVID-19, Public Health and Social Measures (PHSM) strongly recommended by WHO (2021), such as restrictions on public and private gatherings, were conducted in the worldwide. Amid the COVID-19 pandemic, the psychological wellbeing of women in the perinatal period is at stake but often overlooked (1).

Motherhood transition is the challenging period during which women need to acquire various parenting knowledge and skills, adapt to the changing family relationships, and accept the maternal role (2). A great amount of first-time mothers find it too hard to cope with these physical, mental, and social challenges after childbirth because they lack the parenting experience (3, 4). In the initial postpartum period, numerous primiparous women have been reported to frequently encounter various kinds of parenting problems, i.e., unsuccessful parenting tasks and poor maternal role transition (3, 4), which have obviously detrimental consequences on maternal wellbeing and infant development (5–7).

As a significant predictor of parenting outcomes, the maternal self-efficacy (MSE) is defined as the beliefs of women having about their capability of the organization and performance of various parenting tasks (8). Research found that compared with mothers in developed countries (9–11), Chinese first-time mother had a lower MSE level, and especially had fewer confidence in common diseases management and emergency care of infants (2, 6, 12). Some factors have been identified in the existing literature to affect MSE, and the main factors influencing MSE are postpartum depression (PPD) and social support (6, 13, 14).

Previous studies indicated that a larger proportion of Chinese primiparous women appeared to suffer from PPD in comparison of women in Western countries, because Chinese mothers in the traditional culture had been given a high expectation of the maternal role, and had to cope with the delicate relationship with mothers-in-law (12, 15). It needs to be noted that the results from the recent meta-analysis found the pooled prevalence of PPD among postpartum women during the COVID-19 pandemic was much higher than the incidents of previous research during non-pandemic period (1, 16), which indicated the COVID-19 pandemic could detrimentally affect mental wellbeing of postpartum women after delivery.

In regarding to social support, Chinese primiparous women were reported to acquire insufficient social support after childbirth, and particularly lack adequate informational support and evaluative support from health professionals, such as professional parenting advice and instructions (2, 12). In particular, various supports during the pandemic were seriously limited owing to the restrictions that have been undertaken to decrease the risk of transmission of COVID-19 (17). The limited access to routine maternity care during the COVID-19 pandemic was likely to further exacerbate poor mental health and parenting confidence of women (18). Therefore, the tailored interventions from health professionals and policy makers could be offered to improve the parenting and mental health outcomes during the COVID-19 pandemic.

Before pandemic, some traditional face-to-face interventions were proved to be effective in the improvement of maternal parenting and mental wellbeing (15, 19, 20). However, the feasibility and generalizability of the traditional interventions were hindered by some factors especially during the pandemic period. Firstly, the shortage of health professionals and the larger numbers of postpartum women in China could negatively affect the accessibility of the face-to-face health intervention (21, 22). Secondly, the stigma on PPD blocked many new mothers seeking face-to-face intervention, which was particularly prominent in developing countries (23). Thirdly, the time and financial constraints and the struggles with parenting a baby were the other barrier for primiparous women to seek traditionally face-to-face professional help (21). Fourthly, the social isolation and health service disruptions caused by the pandemic seriously decreased the feasibility of the face-to-face intervention. Thus, these barriers indicated the urgent need for new form of intervention to improve maternal parenting and mental health outcomes during the COVID-19 pandemic.

Research found that internet interventions in health field can contain more tailor information, reach larger groups of participants, provide more anonymity, and reduce financial and time cost in comparison with face-to-face interventions (24). In recent years, some internet-based interventions on mental health outcome were conducted during the COVID-19 pandemic. For instance, one randomized controlled trial (RCT) aimed to determine the effect of an online psychoeducational support on the perceived stress of caregivers of COVID-19 survivors, and found that after receiving a psychoeducational support training program for stress management through six online group sessions, cases in the intervention group had a greater decrease in the perceived stress in comparison with cases in the control group (25). The other internet-based intervention was verified to effectively address psychological distress for adults with at least mild depressive symptoms (26). Furthermore, some Portuguese psychologists used the digital information and communication technologies (ICTs) in psychological counseling during the pandemic, and described that their experience with the use of ICTs was positive, meeting clients' adherence and yielding good results (27).

At present, approximate two-thirds of Chinese people were reported to access the internet *via* a device of mobile or computer (28). Therefore, the internet-based support program (ISP) was firstly designed to improve Chinese primiparous women's parenting ability, mental wellbeing, and social support; and to our knowledge, this is the first rigorous designed RCT based on internet platform on parenting and mental health outcomes for Chinese first-time mothers during the COVID-19 pandemic (13). Our pilot study identified that the ISP could significantly increase primiparous women's parenting and mental wellbeing; however, the pilot study findings were restricted by the small sample (*n* = 44) (29). Thereby, the ISP was further investigated in the present research with a larger sample to confirm whether it could be adopted as a significant source for primiparous women to improve their parenting confidence and mental wellbeing during the COVID-19 pandemic.

## Materials and methods

### Study design

A multi-center RCT was conducted to evaluate the effectiveness of an internet-based support program (ISP) on the improvements of MSE, social support; and the alleviation of PPD symptoms for Chinese primiparous women during the COVID-19 pandemic. The study was strictly adhered to the guidelines of the Declaration of Helsinki, and approved by the Ethics Committee of Medical School, X University (Approval number: 2020011). The trial protocol was published in January (13).

### Participants, randomization, and blinding

The participants were recruited in the maternity wards of two tertiary public hospitals (about annual birth of 4,000 per hospital) in Shenzhen City, Guangdong Province of China. The inclusion criteria are: (1) primiarous women with healthy babies; (2) ≥18 years old; (3) married; (4) having ability to response; (5) being available to the internet. The exclusion criteria are: (1) women having depression history; (2) with a serious physical or mental condition of either the mother or the infant; (3) women undergoing any other physical or psychological intervention.

The information sheets were distributed to all eligible women to introduce the purposes and process of the clinical trial. The participants were informed of freedom to withdraw at any time and were assured of confidentiality by using special code numbers to identify themselves. The informed consent was obtained from every participant before data collection. With a power of 0.80, an alpha set at 0.05 and an effect size of 0.35 for the outcome of PPD (EPDS scores), each group was 90 women (15). Assuming an attrition rate of about 30% according to the non-response rate of 9–29% in previous research (13), a minimum of 226 women (113 in each group) were required in the study.

**Figure 1:**
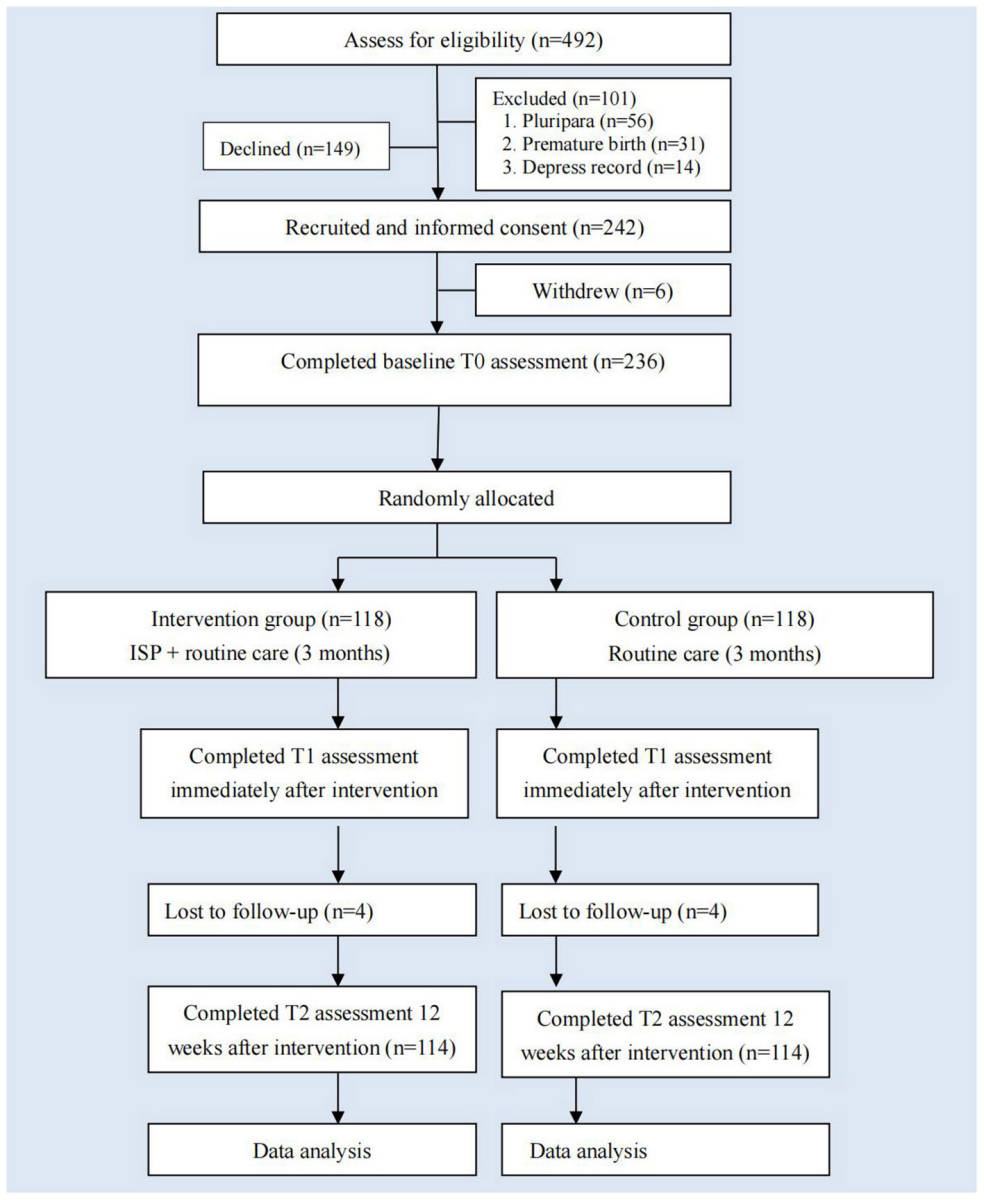
Consolidated Standard of Reporting Trials (CONSORT) flowchart in the research. SICS, the Self-efficacy in Infant Care Scale; EPDS, Edinburgh Postnatal Depression Scale; PSSS, Postnatal Social Support Scale.

From May 2020 to March 2021, a total of 492 primiparous women were approached. Of which, 149 women were declined to participant, and 101 women were excluded as they did not meet the inclusion criteria. Finally, 242 participants were recruited in the study. The Consolidated Standards of Reporting Trials (CONSORT) (30) flowchart is illustrated in Figure 1. The participants were randomly assigned (blocked randomization, allocation 1:1) to the intervention group and the control group. Sequence randomization was performed using a computerized random number generator, and was kept in closed and opaque envelopes and saved by the researchers who have no direct relationship with the project to make sure that the random allocation was masked in advance. During the research process, the group allocation was blinded to the participants, enrolling researchers, outcome assessors, and data analysts.

### Intervention

The ISP was designed by the theoretical framework combined self-efficacy theory (31) with social exchange theory (32). The other details of ISP contents were described in the published trial protocol (13). Primiparous women in the control group received the routine postpartum care; and participants in the intervention group accessed to the ISP intervention and received the routine postpartum care. Before the COVID-19 outbreak, the routine postpartum care used to include health care from obstetricians and obstetric nurses during the hospitalization of 3–5 days; and about four times of home visiting from community health workers during the first month postpartum (5). By contrast, during the COIVD-19 pandemic, some face-to-face postpartum home visits were suspended, and the alternative online consultation and guidance were provided from community health workers through WeChat, phone or video (33).

**Figure 2:**
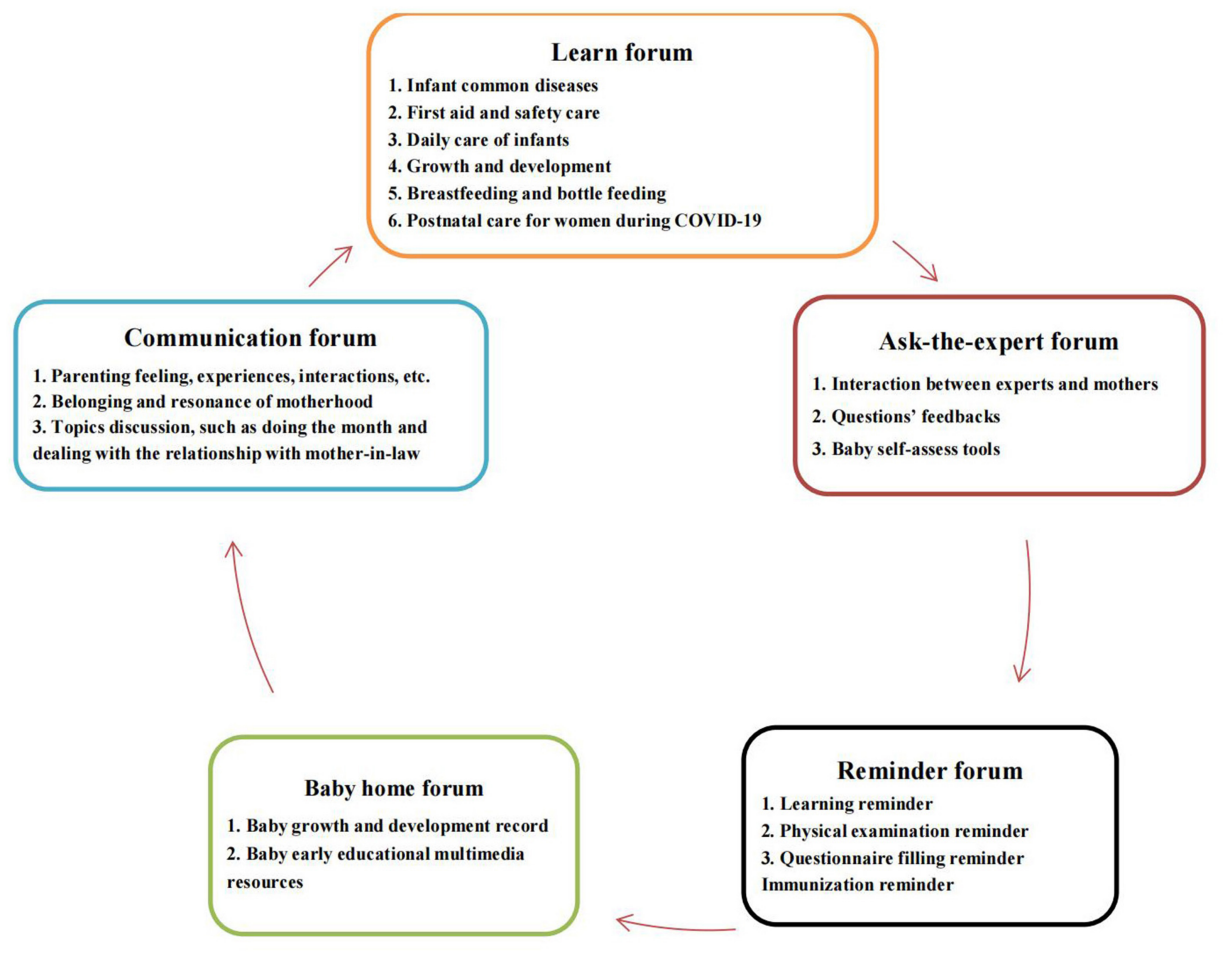
Contents of the ISP.

The components of ISP shown in Figure 2 included learning forum, communication forum, ask-the-expert forum, baby home forum, and reminder forum. The intervention implemented by ISP was last no < 3 months. Women in the intervention group were encouraged to log in the ISP whenever they were available and to learn parenting knowledge and skills in learning forum, to acquire much more parenting experience and peer supports in communication forum, to received professional assistance in ask-the-expert forum, and to share their parenting story in baby home form. They were asked to connect to the ISP at least twice 1 week, and no less than total 1 h per week. Their frequency and duration of the ISP logins were monitored in the reminder forum to evaluate the women's adherence. Telephone or WeChat reminders were likewise sent to participants to motivate and encourage their engagement in the ISP every week. In the current research, all women in the intervention group met our minimum requirement of logging in the ISP. Intervention outcomes were measured through questionnaires at baseline before randomization (T0, about 3–5 days following birth), post-intervention (immediately after the intervention, T1, about 3 months postpartum), and three-month follow up (T2, about 6 months postpartum).

### Outcomes

#### Primary outcome

Maternal self-efficacy (MSE) as the primary outcome was comparing between the intervention group and the control group at T0, T1, and T2. The Self-efficacy in Infant Care Scale (SICS) was used to measure MSE (34). This tool has 44-items, and each item refers to one parenting task. Women are supposed to assess their belief of confidence in performing various parenting tasks from “Not confident at all to do it” (0 point) to “Definitely confident I can do it” (100 point). The scale is scored by summing the numerical ratings and dividing by the number of tasks. The higher average score women acquire indicates the higher MSE level women have. The Cronbach's alpha coefficient of SICS was 0.96, and the test–retest reliability coefficient of SICS was 0.93. The internal consistency of Chinese version of SICS was 0.95 (29). In the present study, the internal consistency of the SICS was positive (Cronbach's alpha = 0.96).

Women's postpartum depression (PPD) was measured likewise as a primary outcome at T0, T1, and T2. The Edinburgh Postnatal Depression Scale (EPDS) was used to evaluate PPD (35). The 10-item self-reported scale is based on a four-point Likert score [0–3], and the total score of EPDS ranges between 0 and 30. The lower score women acquire means the better mental health status women have. The reported Cronbach's alpha coefficient of Chinese version EPDS was 0.87; and the concurrent validity with the Beck Depression Inventory (BDI) was 0.79 (36).The Cronbach's alpha coefficient of the EPDS was 0.84 in this study.

#### Secondary outcomes

Social support was measured as the secondary outcome at T0, T1, and T2. The Chinese version of Postnatal Social Support Scale (PSSS) was used to measure Chinese women's perception of received support after delivery (37). This 20-item tool uses a four Likert-type point, with each item being scored from 0 to 3. The total score of PSSS ranges from 0 to 60 points, with the higher score indicating the more social support women receiving. The reported Cronbach's alpha coefficient for Chinese version of PSSS was 0.89, and the test–retest reliability coefficient of this tool was 0.92 (37). In the current research, the Cronbach's alpha coefficient of the PSSS was 0.90.

#### Other outcomes

Women self-reported their social-demographic and clinical variables at T0, including the data of maternal age, educational level, occupation, family income per month, mode of childbirth, whether attending parenting training before delivery, baby gender, baby health and baby fussiness. The medical records were checked if doubts existed regarding the clinical variables.

### Data collection

The baseline assessment at T0 was undertaken by the researchers, and participants were asked to complete the SICS, EPDS, PSSS and social-demographic and clinical data in the maternity wards. Post-intervention assessment at T1 was conducted immediately after the intervention, and follow-up assessment at T2 was carried out on 3 months after the intervention. The electronically questionnaires comprised of SICS, EPDS, and PSSS were sent to participants by WeChat or email at the two time points of T1 and T2; and the completed questionnaires of T1 and T2 were returned to the researchers likewise by WeChat or email. In order to decrease the non-response rate, a reminder telephone or WeChat was given to participants before and after 1 week of the two time points, respectively. All collected data were kept confidentially.

### Data analysis

All the statistical analyses were conducted by the Statistical Package for Social Sciences (SPSS, 20.0). Descriptive analysis was undertaken to describe the social-demographic and clinical data. Mean and standard deviation (SD) were used for normally distributed data, and medians and interquartile ranges (IQRs) were used for data that are not normally distributed; frequencies and percentages were used for categorical data. A two-tailed *p* < 0.05 can be considered as statistically significant. The chi-square (χ^2^) for categorical variables and the independent sample *t*-test for continuous variables were conducted to detect any significant difference between the intervention group and the control group on the social-demographic characteristics and baseline outcomes. The effects of intervention on the improvements of MSE, and social support; and the alleviation of PPD symptoms across the three time points were evaluated by the repeated measures multivariate analysis of covariance to explore how outcomes has changed between groups; over time, and the interaction between group and time.

## Results

### Participant characteristics

Between May 2020 and March 2021, 492 primiparous women were assessed for eligibility: 149 women (149/492, 30.3%) refused, and 101 women (101/492, 20.5%) were ineligible, and 242 participants (242/492, 49.2%) were recruited in the study. Of which, 236 women completed baseline T0 assessment and underwent random assignment. Finally, 114 women in the intervention group and 114 women in the control group completed the follow-up measurement.

The mean age of these participants was 27.30 (3.00) years. Of which, 44.1% (104/236) of respondents had a University degree, and 41.5% of them (98/236) had a professional occupation. More than half of women (122/236, 51.7%) had a family income of >5000yuan (US$700)/per month, person. Research found that there was no significant difference between the intervention and the control groups regarding social-demographic, clinical-related, and baseline outcome measures at T0 (Table 1).

**Table 1:** Comparison of socio-demographic and clinical characteristics and baseline outcomes between the groups.

**Variables**	**Total (*n =* 236)**	**Intervention group (*n =* 118)**	**Control group (*n =* 118)**	***t*/X^2^ value**	***p-*value^a^**
**Maternal age, mean (SD)**	27.35 (3.00)	27.28 (3.10)	27.42 (2.93)	−0.346	0.730
**Educational level**, ***n*** **(%)**				1.619	0.445
Middle school or lower	47 (19.9)	27 (22.9)	20 (16.9)		
High school	85 (36.0)	39 (33.1)	46 (39.0)		
University/College or higher	104 (44.1)	52 (44.0)	52 (44.1)		
**Occupation**, ***n*** **(%)**				2.824	0.419
Professional	98 (41.5)	52 (44.1)	46 (39.0)		
Skilled	42 (17.8)	18 (15.3)	24 (20.3)		
Unskilled	60 (25.4)	33 (28.0)	27 (22.9)		
Unemployed	36 (15.3)	15 (12.7)	21 (17.8)		
**Family income per month**, ***n*** **(%)**				4.283	0.117
< 3000 yuan (US$420)	23 (9.7)	15 (12.7)	8 (6.8)		
3001-−5000 yuan (US$420–700)	91 (38.6)	39 (33.1)	52 (44.1)		
>5000 yuan (US$700)	122 (51.7)	64 (54.2)	58 (49.2)		
**Mode of Childbirth**, ***n*** **(%)**				1.928	0.381
Natural childbirth	135 (57.2)	72 (61.0)	63 (53.4)		
Assisted childbirth	61 (25.8)	26 (22.0)	35 (29.7)		
C-section	40 (17.0)	20 (16.9)	20 (16.9)		
**Whether attending parenting train**, ***n*** **(%)**				0.000	1.000
Yes	104 (44.1)	52 (44.1)	52 (44.1)		
No	132 (55.9)	66 (55.9)	66 (55.9)		
**Baby gender**, ***n*** **(%)**				0.100	0.752
Boy	120 (46.2)	59 (50.0)	61 (51.7)		
Girl	116 (44.6)	59 (50.0)	57 (48.3)		
**Baby health, mean (SD)**	61.69 (18.32)	61.98 (16.59)	61.39 (19.96)	0.248	0.804
**Baby fussiness, mean (SD)**	49.92 (12.63)	50.97 (12.36)	48.88 (12.86)	1.270	0.206
**Outcomes, mean (SD)**					
SICS	63.59 (7.02)	63.55 (6.95)	63.63 (7.12)	−0.093	0.926
EPDS	5.18 (2.28)	5.14 (2.43)	5.22 (2.13)	−0.256	0.798
PSSS	41.42 (2.79)	41.42 (2.74)	41.42 (2.84)	−0.019	0.985

a All p-values were calculated using independent samples t test for continuous variables and chi-square tests or Fisher exact test for categorical variables. SICS, Self-efficacy in Infant Care Scale; EPDS, Edinburgh Postnatal Depression Scale; PSSS, Postpartum Social Support Scale.

### Intervention outcomes

Compared with first-time mothers in the control group, primiparous women in the intervention group were found to have a higher SICS score at T1 (mean difference = 6.66, 95% CI: 4.88–8.45, *p* < 0.001), and T2 (mean difference = 5.88, 95% CI: 4.08–7.67, *p* < 0.001); a lower EPDS score at T1 (mean difference = −3.05, 95% CI: −3.80 to −2.30, *p* < 0.001), and T2 (mean difference = −2.46, 95% CI: −3.09 to −1.82, *p* < 0.001); and a higher PSSS score at T1 (mean difference=4.27, 95% CI: 3.41–5.13, *p* < 0.001); but no significant difference at T2 (mean difference = 0.34, 95% CI: −0.46 to 1.14, *p* = 0.41) (Table 2).

**Table 2:** Effect of ISP on outcomes of parenting, mental health, and social support at T1 and T2.

**ISP effect**	**Mean (SD) Intervention group (*n =* 114)**	**Mean (SD) Control group (*n =* 114)**	**Mean difference (95% CI)**	***p*-value^a^**
**Primary outcomes**				
**MSE (SICS)**				
T1	73.53 (6.21)	66.86 (7.40)	6.66 (4.88 to 8.45)	< 0.001
T2	72.90 (6.73)	67.02 (7.01)	5.88 (4.08 to 7.67)	< 0.001
**Secondary outcomes**				
**Postpartum depression (EPDS)**				
T1	6.03 (2.50)	9.08 (3.20)	−3.05 (−3.80 to −2.30)	< 0.001
T2	5.70 (2.23)	8.16 (2.60)	−2.46 (−3.09 to −1.82)	< 0.001
**Social support (PSSS)**				
T1	45.70 (3.73)	41.42 (2.82)	4.27 (3.41 to 5.13)	< 0.001
T2	42.90 (3.29)	42.56 (2.83)	0.34 (−0.46 to 1.14)	0.405

a All p-values were calculated using repeated measures multivariate analysis of covariance. ISP, Internet-based support program; SICS, Self-efficacy in Infant Care Scale; EPDS, Edinburgh Postnatal Depression Scale; PSSS, Postpartum Social Support Scale; T1, immediately after the intervention; T2, three-month follow.

**Figure 3:**
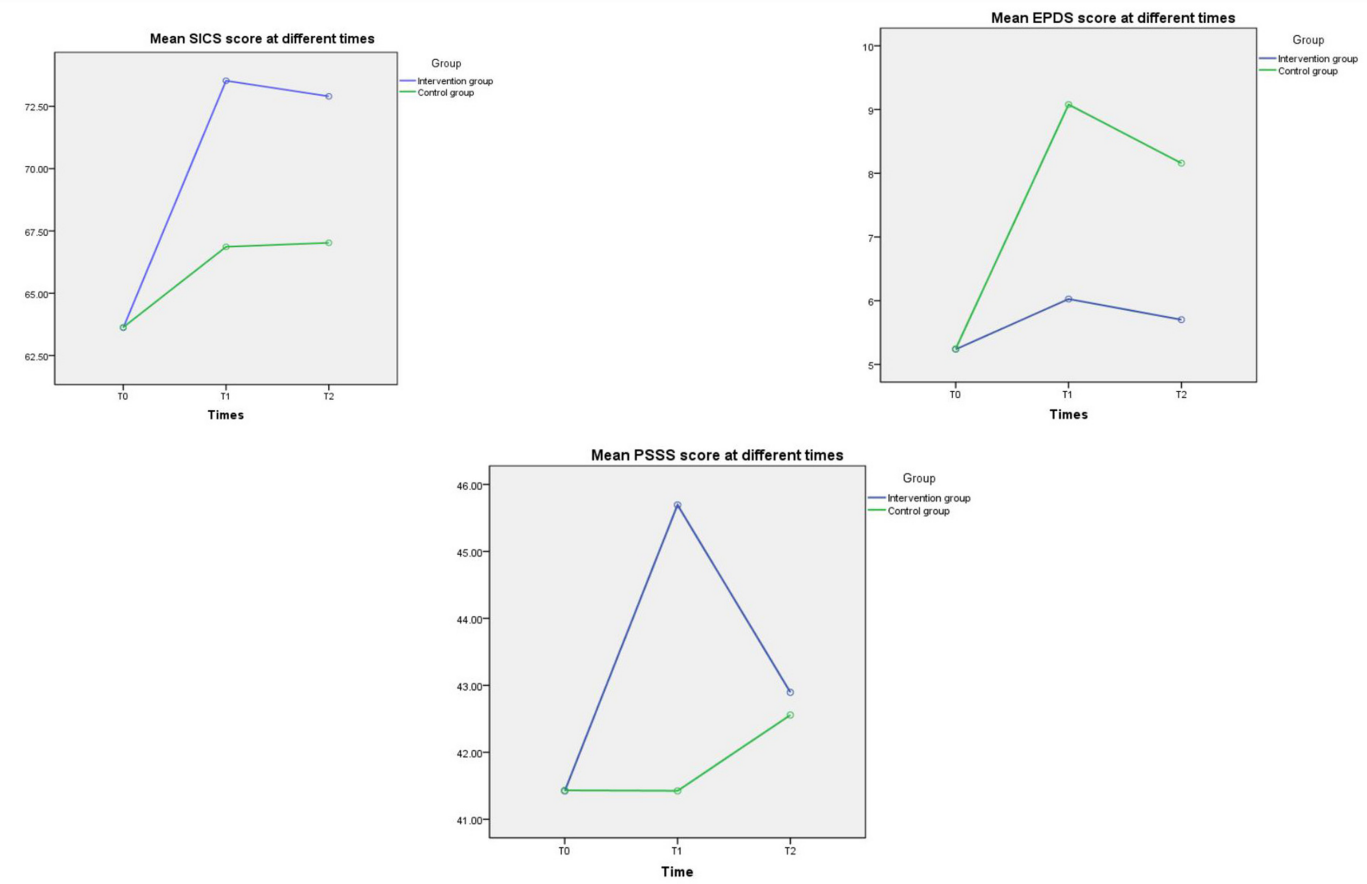
The mean score change in SICS, EPDS and PSSS at T0, T1, and T2. SICS, the Self-efficacy in Infant Care Scale; EPDS, Edinburgh Postnatal Depression Scale; PSSS, Postnatal Social Support Scale.

Our research found that the mean SICS score of these participants significantly increased from T0 to T1 (mean difference = 9.91, 95% CI: 9.14–10.69, *p* < 0.001), T0 to T2 (mean difference = 9.29, 95% CI: 8.47–10.10, *p* < 0.001) in both the intervention group and the control group (mean difference of T0 to T1 = 3.23, 95% CI: 2.45–4.00, *p* < 0.001; mean difference of T0 to T2 = 3.39, 95% CI: 2.58–4.20, *p* < 0.001); however, the increase was much higher for women in the intervention group than women in the control group. No significant difference of EPDS score was found in the intervention group with the passage of time (mean difference of T0 to T2 = 0.47, 95% CI: 0.00–0.93, *p* = 0.05). By contrast, the significant increased EPDS score was found in the control group from T0 to T1 (mean difference = 3.83, 95% CI: 3.28–4.39, *p* < 0.001), T0 to T2 (mean difference = 2.91, 95% CI: 2.45–3.38, *p* < 0.001); and the significant decreased EPDS score was found in the control group from T1 to T2 (mean difference = −0.92, 95% CI: −0.53 to −1.31, *p* < 0.001). The PSSS score of women in the intervention group was shown to have a significant increase from T0 to T1 (mean difference = 4.27, 95% CI: 3.66–4.88, *p* < 0.001), T0 to T2 (mean difference = 1.47, 95% CI: 1.13–1.82, *p* < 0.001); while, no such increase in social support score was found in the control group with the passage of time (mean different of T0 to T1 = 0.01, 95% CI: −0.60 to 0.62, *p* > 0.99). The mean score change in SICS, EPDS and PSSS at the different time points of T0, T1, and T2 was illustrated in Figure 3.

### Association between the frequency and duration of the ISP logins and the intervention outcomes

In the current research, the usage of the ISP was varied from every primiparous woman in the intervention group. During the 3 months period of intervention, the frequency of the ISP logins ranged from 24 to 504 times (median: 81, IQR: 50–278), and the correlation between the frequency of the ISP logins and intervention outcomes was not observed. The duration of the ISP logins ranged from 756 to 4,428 min (median: 1,907, IQR: 834–2,769), and the association between the ISP usage duration and intervention outcomes was found at T1 and T2. To be more specific, MSE (*r* = 0.421, *p* < 0.001 at T1, *r* = 0.321, *p* = 0.02 at T2) and social support (*r* = 0.313, *p* = 0.01 at T1, *r* = 0.297, *p* = 0.03 at T2) had a positive correlation with the ISP usage duration; however, postpartum depression (*r* = 0.356, *p* = 0.02 at T1, *r* = 0.283, *p* = 0.04 at T2) was negatively related with the IPS usage duration at the two time points.

## Discussion

This multi-center RCT aimed to evaluate the effectiveness of an internet-based support program (ISP) on the improvements of MSE, social support; and the alleviation of PPD symptoms for Chinese primiparous women during the COVID-19 pandemic. Our research found that compared with first-time mothers in the control group, primiparous women in the intervention group had a significant higher level of MSE at T1 and T2, a higher level of social support at T1; and experienced less PPD symptoms at T1 and T2, which was consistent with our pilot study results (29). The research findings identified that as an effective and easily accessible intervention, the ISP could become the new choice for health professionals to support primiparous women on parenting outcomes and mental wellbeing after childbirth.

To be specific, the ISP was found to increase the MSE level for Chinese first-time mothers, which was well aligned with the findings of prior research that were *via* face-to-face intervention approach (15, 19, 20); and the positive outcomes of ISP on maternal parenting can achieve a long-term effect of 3 months. Chinese primiparous women were reported to have a lower MSE level, and frequently suffered from various parenting problems that negatively affected maternal wellbeing and infant development (5–7). Therefore, the tailored online intervention of ISP was designed to increase their parenting capability for these first-time mothers. According to the self-efficacy theory (30) and the social exchange theory (32), the sharing parenting learning materials and experiences, and the various kinds of supports can effectively improve new mothers' parenting ability and confidence. So that, the contents of ISP were designed to incorporate various parenting knowledge and skills, especially about common diseases management and emergency care of infants in the learning forum; the sharing parenting feeling and experience in the communication forum and baby home forum; the professional parenting advice and instructions in the ask-the-expert forum; and the kindly suggestions offered from other mothers in the communication forum. Moreover, our research found that the mean MSE score of these participants significantly increased from T0 to T1, T2 in both the intervention group and the control group. It indicated that as time went on, all participants were prone to be familiar with various parenting tasks and adapt to the new maternal role, then have a higher level of parenting confidence. The consistent results were likewise reported by the previous studies (6, 38, 39). Interestingly, the women in the intervention group was found to experience significantly greater increase of MSE than women in the control group; and the result likewise strongly verified the effect of ISP on the improvement of MSE levels.

Owing to the high prevalence and detrimental consequences, PPD has been identified as one of the serious global public health issues in the last decade (40). Especially during the COVID-19 pandemic, the higher incidence of PPD among postpartum women was reported compared with the prevalence of PPD before pandemic (1). Therefore, the targeted intervention strategy was in urgent need to prevent long-term impacts of the COVID-19 pandemic on maternal mental wellbeing (41). Fortunately, our ISP was identified to significantly alleviate PPD symptoms for Chinese primiparous women during the COVID-19 pandemic; and the positive outcome on mental wellbeing was proved to last 3 months after intervention. In order to improve the mental wellbeing of new mothers, the ISP was designed to promote women's capability to successfully fulfill various parenting tasks, to teach women how to initiatively mediate their mood, to help them positively cope with the relationship with mother-in-law, and to alleviate women' s fears experienced during the COVID-19 pandemic (13). Moreover, our research found that women in the control group experienced a significant increase in the score of EPDS from T0 to T1 (mean difference = 3.883, *p* < 0.001), and the significant decrease in the score of EPDS from T1 to T2 (mean difference = −0.921, *p* < 0.001). It was aligned with the previous observational research, in which women were reported to have a peak incidence of PPD at approximately 6–8 weeks after childbirth, and the symptoms of PPD could alleviate with the passage of time (42, 43). However, this kind of PPD remittance as time went on without any intervention strategy was too little to have clinical significance (5, 6). Therefore, the ISP was highly needed to promote primiparous women's mental wellbeing in the early stage of motherhood.

Owing to decrease the risk of transmission of COVID-19, delivering psychosocial support was seriously limited during the pandemic (17). Thus, special support measures from health professionals should be conducted to improve the maternal and infant outcomes during the lockdown period. Our research reported that compared with first-time mothers in the control group, primiparous women in the intervention group had a higher social support score at T1 (mean difference = 4.27, *p* < 0.001); while no significant difference at T2 (mean difference = 0.34, *p* = 0.74). The research findings indicated that the ISP could improve social support level for first-time mothers in short term; however, the significant outcomes on social support did not achieve the long-term effect, which was inconsistent with the previous research (15, 19). It was possible that women in the study had accessed the ISP only for 3 months, and the effects of ISP on social support could cause little residual advantage at 6 months postpartum of T2 time point. Thereby, suggesting women to retain the ISP access in longer-term may lead to different social support outcomes (44), which needs to be addressed in the future research.

This study possessed several limitations. Firstly, owing to the nature of the research, blinding of researchers during the whole research process can be impossible, which may cause the potential biases. Secondly, the requirement of internet access may have resulted in a more tech-savvy population recruited in the study, potentially limiting the generalization of this study. Thirdly, the self-report tools were used to measure the main variables, such as PPD, and may cause social desirability bias owing to the traditional belief of “domestic shame should not be made public”. Fourthly, because of time and financial limitations, the outcomes of ISP only focused on Chinese primiparous women. The knowledge gained from this study are strongly recommended to be used to plan a culturally appropriate ISP for other kinds of postpartum women, such as first-time mothers from different countries, single mothers, and multiparas.

## Conclusion

The effect of our ISP was evaluated to significantly increase the levels of MSE, social support, and to alleviate PPD symptoms for Chinese first-time mothers after childbirth during the infectious disease outbreak. This research is unique in its contribution to the new online intervention to support primiparous women on parenting and mental health outcomes. As an effective and easily accessible intervention, ISP could become a significant source for health professionals to support primiparous women on parenting outcomes and mental wellbeing in the postpartum period especially during the COVID-19 pandemic.

## Data availability statement

The data presented in this study are available on request from the corresponding author. The data are not publicly available due to privacy restrictions.

## Ethics statement

The studies involving human participants were reviewed and approved by the Ethics Committee of Medical School, Shenzhen University (Approval number: 2020011). The patients/participants provided their written informed consent to participate in this study.

## Author contributions

Conceptualization, project administration, and funding acquisition: XZ. Methodology: XZ and JZ. Investigation: SL, LH, QF, and XZ. Data analysis and writing—original draft preparation: XZ, YZ, JZ, and SL. Writing—review and editing: XZ and YZ. All authors contributed to the article and approved the submitted version.
